# Attention Biases for Eating Disorder-Related Stimuli Versus Social Stimuli in Adolescents with Anorexia Nervosa – An Eye-Tracking Study

**DOI:** 10.1007/s10802-022-00993-3

**Published:** 2022-11-23

**Authors:** Anca Sfärlea, Anne Kathrin Radix, Gerd Schulte-Körne, Tanja Legenbauer, Belinda Platt

**Affiliations:** 1 Department of Child and Adolescent Psychiatry, Psychosomatics and Psychotherapy, University Hospital, LMU Munich, Research Department, Nussbaumstr. 5, 80336 Munich, Germany; 2 LWL-University Clinic of the RUB Bochum, Department of Child and Adolescent Psychiatry, Psychotherapy, Psychosomatics, Hamm, Germany

**Keywords:** Anorexia nervosa, Adolescence, Attention bias, Emotional faces, Body pictures

## Abstract

Anorexia nervosa (AN) is characterized by attention biases for eating disorder-related information as well as altered attentional processing of social information. However, little is known about the interplay between the altered attentional processing of these two types of information. The present study investigates attention biases for eating disorder-related information (pictures of bodies) versus social information (pictures of faces), in adolescents with AN. Attention biases were assessed via eye-tracking during a passive-viewing task in which female bodies and faces were presented simultaneously and thus competed directly for attention. Female adolescents (13–18 years) with AN (*n* = 28) were compared to a clinical comparison group (adolescents with major depression; *n* = 20) and a comparison group of adolescents with no mental illness (*n* = 24). All groups looked longer at bodies than at faces, i.e., showed attention biases for bodies in maintenance of attention. These biases were more pronounced in adolescents with AN than in both comparison groups, particularly for underweight bodies, at the expense of looking less at social stimuli. The results indicate “dual” attention biases in adolescents with AN (i.e., towards bodies and away from emotional faces) which could have a twofold negative impact on eating disorder psychopathology: increased attention to eating disorder-related information might directly influence eating disorder symptoms while less attention to social information might have an indirect influence through the amplification of interpersonal difficulties.

**Supplementary Information:**

The online version contains supplementary material available at 10.1007/s10802-022-00993-3.

## Introduction

Anorexia nervosa (AN) is a severe mental disorder that mostly affects adolescent and young adult women with an onset age peak at 15.5 years (Solmi et al., 2022). It has a relatively poor long-term prognosis: Only between one and two thirds of patients recover fully while up to 30% of cases take a chronic course (Eddy et al., 2017; Fichter et al., 2017; Herpertz-Dahlmann et al., 2018; Rydberg Dobrescu et al., 2020), resulting in the highest mortality rate of all mental disorders (Arcelus et al., 2011; Harris & Barraclough, 1998). This underlines the need for a deeper understanding of the mechanisms involved in the etiology and maintenance of the disorder.

AN is characterized by significantly low body weight, intense fear of gaining weight and body image disturbance (American Psychiatric Association, 2013). These core symptoms are accompanied by dysfunctional cognitions about food, weight, and shape (Vitousek & Hollon, 1990; Williamson et al., 1999, 2004), including attention biases for eating disorder (ED) -related information like pictures of food and bodies (Lee & Shafran, 2004; Ralph-Nearman et al., 2019; Stott et al., 2021). Attention biases are defined as automatic tendencies to preferentially attend to information that is consistent with one’s (maladaptive) cognitive schemata, for example disorder-related or negative information (Aspen et al., 2013). Importantly, biases for disorder-related information are proposed to not only represent an epiphenomenon of AN but to play a role in the development and maintenance of the ED (Aspen et al., 2013; Vitousek & Hollon, 1990; Williamson et al., 1999, 2004).

In addition to difficulties related to eating and body image, individuals with AN have also been found to show difficulties in social and emotional functioning (Caglar-Nazali et al., 2014; Mason et al., 2021; Oldershaw et al., 2011; Tauro et al., 2022) and these difficulties have also been proposed to contribute to the disorder’s development and maintenance (Treasure & Cardi, 2017; Treasure et al., 2012). For example, individuals with AN have been found to show alterations in the neurophysiological correlates of attentional processing of social stimuli such as faces (Fonville et al., 2014; Sfärlea et al., 2016), indicating, among other things, that they might show reduced selective attention for other people’s faces and perceive them as less intrinsically salient, i.e., less relevant for themselves (Sfärlea et al., 2016). Such alterations in the attentional processing of important social cues could contribute to individuals with AN increasingly isolating themselves from their family and peers, and this, in turn, might not only foster interpersonal problems but also exacerbate ED symptoms as a maladaptive reaction to these problems (Treasure et al., 2012).

Attention biases for social information, such as emotional faces, have been proposed as another factor contributing to socio-emotional difficulties in individuals with EDs (Harrison et al., 2010a). Research on such biases in individuals with AN have yielded mixed results: While some studies found stronger attention biases towards faces showing negative emotions in individuals with AN compared to those with no mental illness (Cardi et al., 2013; Harrison et al., 2010a, b), others found individuals with AN to turn their attention away from negative faces (Kim et al., 2014), and yet others found no differences in attention biases for emotional faces between individuals with AN and individuals with no mental illness (Bang et al., 2017; Cardi et al., 2015; Goddard & Treasure, 2013; Schneier et al., 2016). Most of these studies have investigated attention biases for angry faces as anger is suggested to play an important role in ED psychopathology (Ioannou & Fox, 2009). Attention biases *towards* angry faces have been suggested to reflect anger being particularly salient for individuals with AN (Harrison et al., 2010a) while attention biases *away from* angry faces, i.e., avoidance of angry faces, has been suggested to reflect anger being perceived as “toxic” and unacceptable by individuals with AN (Geller et al., 2000; Kim et al., 2014). Attention biases away from angry faces have been found to be associated with social communication difficulties in youth with autism spectrum disorders (García-Blanco et al., 2017) as they may involve behavioral avoidance of potentially aversive social interactions and thus impede the ability to adaptively solve interpersonal conflicts (García-Blanco et al., 2017).

To date, attention biases for disorder-relevant information and social information in AN have mostly been investigated as two different lines of research. Few studies have assessed attention biases for ED-related stimuli in direct comparison to social stimuli, i.e., have presented ED-related and social stimuli simultaneously so that these stimuli compete for attention. Investigating attention biases for disorder-relevant information *versus* social information might be a key to understanding how different factors involved in the maintenance of the disorder relate to each other. It could help to understand, for example, the altered processing of social cues in AN: Preoccupation with weight and shape, reflected in disorder-related information automatically attracting more attention than social information, might explain why social information like emotional faces seems to be less salient for individuals with AN (Sfärlea et al., 2016). The first study to examine attention to disorder-related versus social information (Watson et al., 2010) recorded eye-movements while participants viewed either pictures of faces or whole-body pictures including faces. When bodies were presented together with faces, weight-recovered women with AN spent less time looking at the faces compared to women without EDs, thus showing an attention bias towards bodies. Pinhas et al. (2014) also used eye-tracking and presented pictures of bodies and pictures of social interactions simultaneously. Adolescents with AN spent more time looking at body pictures than at pictures of positive social interactions, while this was not the case in adolescents with no mental illness. When thin and overweight body pictures were both presented alongside pictures of social interactions, adolescents with AN preferentially looked at the thin body pictures.

The aim of the present study was to add to this emerging body of literature by investigating attention biases for disorder-relevant information, i.e., pictures of bodies, versus social information, i.e., pictures of faces, in adolescents with AN. We focus on adolescents as adolescence is the most common time for the onset of AN (Somli et al., 2022) while at the same time difficulties in social functioning could have particularly adverse consequences in adolescence due to increasing social demands in this developmental period (Happé & Frith, 2014). When studying adolescents with AN, it has to be taken into account that a high proportion are also affected by comorbid mental disorders, especially depression (Bühren et al., 2014; Jaite et al., 2013). Adolescents with depression have been found to show attention biases for social information like faces (Lau & Waters, 2017; Platt et al., 2017) and it is possible that biases for body-related stimuli are also not a specific characteristic of individuals with EDs but are present transdiagnostically in various mental disorders. Previous studies have not addressed this possibility so we aimed to extend their findings by comparing adolescents with AN not only to a group of adolescents with no mental illness (“healthy” comparison; HC) but also to a clinical comparison group consisting of adolescents with major depression (MD).^1^ This allows to draw conclusions about the specificity of biases for AN.

Most studies investigating attention biases in individuals with AN have used emotional Stoop or modified Dot-Probe tasks (Aspen et al., 2013; Jiang & Vartanian, 2018; Lee & Shafran, 2004) which infer attention biases from differences in reaction times between different experimental conditions. The assessment of attention biases via reaction-time based measures, however, entails several limitations: i) they provide only a snapshot of attention at a single point in time (Armstrong & Olatunji, 2012), not accounting for the fact that attention consists of different distinct and consecutive subprocesses (Jiang & Vartanian, 2018; Kerr-Gaffney et al., 2019), ii) they only provide an indirect measure of attention and can be influenced by non-attention-related processes, such as slowed processing speed or response execution (Armstrong & Olatunji, 2012), and iii) both the Stroop (Dresler et al., 2012; Lee & Shafran, 2004) and the Dot-Probe task (Platt et al., 2022; Vervoort et al., 2021; Waechter et al., 2014) have been found to show very poor reliability and validity. An alternative to reaction-time based measures are eye-tracking paradigms which measure attention more directly as they allow to continuously record the course of visual attention over time. This has the advantage of capturing the dynamic nature of attention and being able to distinguish between different subprocesses as different eye-tracking parameters serve as indicators for different processes (e.g., Kerr-Gaffney et al., 2019). This is of particular interest when studying attention biases in AN as it has been suggested that individuals with AN might show first hypervigilance for and then avoidance of disorder-relevant information (Lee & Shafran, 2004). Furthermore, eye-tracking paradigms have been found to show superior psychometric properties in adults (Lazarov et al., 2019; Waechter et al., 2014) as well as adolescents (Platt et al., 2022).

Therefore, we chose a passive-viewing eye-tracking paradigm similar to that of Pinhas et al. (2014) in which we presented disorder-relevant information and social information simultaneously while recording eye movements. The paradigm included two types of trials: i) neutral trials in which normalweight bodies were presented alongside neutral faces and ii) emotional trials in which under- and overweight bodies were presented together with positive (happy) and negative (angry) faces. Angry faces were used as negative emotional faces in line with most previous studies investigating attention biases for emotional faces in AN (Bang et al., 2017; Goddard & Treasure, 2013; Harrison et al., 2010a, b; Kim et al., 2014; Schneier et al., 2016). We investigated two components of attention: initial orientation of attention, indicated by location of first fixation, as well as maintenance of attention, indicated by dwell time over the duration of the trial. Based on previous studies (Pinhas et al., 2014; Watson et al., 2010), we expected to find adolescents with AN to show attention biases for disorder-relevant stimuli even in the presence of socially relevant stimuli, compared to both comparison groups. Furthermore, we expected this bias to be particularly pronounced for underweight stimuli, i.e., we expected that when underweight and overweight body pictures are presented together with emotional faces, adolescents with AN would preferentially look at underweight bodies (in line with Pinhas et al., 2014).

## Methods

### Participants

The study sample consisted of 72^2^ adolescent females aged 13–18 years: *n* = 28 adolescents with AN, *n* = 20 adolescents with MD and *n* = 24 adolescents with no mental illness (HC group). Adolescents with AN were recruited from two University Departments of Child and Adolescent Psychiatry in Germany (inpatients and outpatients), while the adolescents with MD and adolescents with no mental illness were recruited and tested at just one of these sites. Adolescents with MD were all inpatients. Adolescents with no mental illness were recruited through local advertisements and schools. To rule out that differences between the group with AN and the comparison groups were driven by adolescents with AN being recruited and tested at different recruitment sites, we compared AN patients from the two sites. They differed in depression and ED symptoms but neither in other participant characteristics nor in any of our outcome variables.

Psychiatric diagnoses were assessed in all participants using a standardized, semi-structured clinical interview (Kinder-DIPS; Margraf et al., 2017; Schneider et al., 2017). The Kinder-DIPS is a well-established German diagnostic interview that allows diagnosis of a wide range of psychiatric axis I disorders according to DSM-5 (American Psychiatric Association, 2013). The interview was administered to the adolescent participants by trained interviewers, i.e., study staff with a Bachelor’s or Master’s degree in Psychology who received several hours of training and supervision before conducting and evaluating the interviews. In previous studies using this training procedure we found the interrater-reliability of the Kinder-DIPS to be very good (e.g., accordance rates of 100% for current diagnoses of AN or MD in clinical groups as well as for no lifetime diagnoses in non-clinical groups; Lukas et al., 2022; Sfärlea et al., 2020), in line with Neuschwander et al. (2013), who reported accordance rates of at least 97% for all diagnoses. However, interrater-reliability could not be assessed for the present study. Exclusion criteria for all participants were IQ < 80 (measured via the Zahlen-Verbingungs-Test; Oswald, 2016), neurological disorders, psychotic disorders, bipolar disorder, substance abuse, pregnancy, benzodiazepine intake, and non-corrected visual impairment. Depression symptoms were assessed with the German version of the Beck Depression Inventory-II (BDI-II; Hautzinger et al., 2006; available from 69 of the 72 included participants, Cronbach’s α = 0.96 in our sample.), anxiety symptoms were assessed with the German version of the trait version of the State-Trait Anxiety Inventory (STAI-T; Laux et al., 1981; available from 71 of the 72 included participants, Cronbach’s α = 0.96 in our sample), and eating psychopathology was assessed with the German version of the Eating Disorder Examination – Questionnaire (EDE-Q; Hilbert & Tuschen-Caffier, 2016; available from 71 of the 72 included participants, Cronbach’s α = 0.97 in our sample.). For participants with AN or MD, height and weight were obtained from their physicians while for participants with no mental illness, height and weight were measured in the laboratory.

Adolescents were included in the group with AN if they currently met criteria for AN according to DSM-5 (American Psychiatric Association, 2013) and had a body mass index (BMI) of < 18.5 and below the 25th age-corrected percentile (according to Kromeyer-Hauschild et al., 2001). Sixteen of the included participants with AN fulfilled criteria for at least one comorbid mental disorder, including MD (*n* = 12), anxiety disorders (*n* = 10), and obsessive-compulsive disorder (*n* = 3). Mean illness duration of AN (time since first onset) was 19.62 months (*SD* = 21.14; *Mdn* = 12.50; range 3–96).

Adolescents were included in the group with MD if they currently met criteria for an episode of MD according to DSM-5 (American Psychiatric Association, 2013) and reported no current symptoms or history of EDs. Within the group of adolescents with MD, 15 individuals met criteria for one or more comorbid mental disorders, including anxiety disorders (*n* = 13) and obsessive-compulsive disorder (*n* = 1).

Adolescents were included in the HC group if they did not meet criteria for any current or past axis I disorder as assessed by the Kinder-DIPS.

Participant characteristics are presented in Table 1. As expected, the three groups differed in BMI and BMI-percentile, with adolescents with AN having lower values than adolescents with MD and adolescents with no mental illness. Both clinical groups (adolescents with AN as well as adolescents with MD) reported more depression symptoms and ED pathology than the adolescents with no mental illness but did not differ from each other. Groups differed in trait anxiety scores, with adolescents with MD reporting the highest scores and adolescents with no mental illness reporting the lowest scores. This suggests that the group with MD had similar depression symptoms and even more pronounced anxiety symptoms than the group with AN, confirming its suitability as a clinical comparison group that takes into account both, depression *and* anxiety. Furthermore, groups differed also in age and IQ with the group with AN being slightly younger and having a higher IQ than the group with MD and the group of adolescents with no mental illness.

**Table 1:** Demographic and clinical characteristics of the sample

	**AN**	**MD**	**HC**	ANOVA			post-hoc tests
	*n* = 28	*n* = 20	*n* = 24				
	*M* (*SD*)	*M* (*SD*)	*M* (*SD*)	*F*	*p*	*η*^*2*^	
Age	15.37 (1.36)	16.37 (1.14)	16.43 (1.56)	4.89	0.010	0.12	AN < MD = HC
IQ	110.55 (10.75)	102.60 (11.96)	102.38 (11.86)	4.25	0.018	0.11	AN > MD = HC
BMI	16.41 (1.36)	23.95 (5.96)	21.42 (3.26)	25.66	< 0.001	0.43	AN < MD = HC
BMI-percentile (age-corrected)	6.68 (6.98)	63.85 (31.09)	52.04 (32.50)	35.74	< 0.001	0.51	AN < MD = HC
Eating disorder symptoms (EDE-Q)	2.96 (1.84)	1.96 (1.63)	0.92 (0.93)	11.09	< 0.001	0.25	AN = MD > HC
Depression symptoms (BDI-II)	24.32 (16.14)	32.39 (12.71)	6.13 (5.54)	24.31	< 0.001	0.42	MD = AN > HC
Anxiety symptoms (STAI-T)	52.61 (12.77)	60.45 (12.16)	32.48 (7.66)	36.70	< 0.001	0.52	MD > AN > HC

*AN* anorexia nervosa, *MD* major depression, *HC* “healthy” comparison, *IQ* intelligence quotient, *BMI* body mass index, *EDE-Q* Eating Disorder Examination - Questionnaire, *BDI-II* Beck Depression Inventory II, *STAI-T* trait version of the State-Trait Anxiety Inventory, *M* mean, *SD* standard deviation; post-hoc *t*-test remain significant after Bonferroni-Holm correction for multiple testing (Holm, 1979).

Four participants with AN and six participants with MD were receiving psychotropic medication. As psychotropic medication may influence eye-movements (e.g., Reilly et al., 2008; Wells et al., 2014) all analyses were repeated excluding these participants. The overall pattern of results remained the same, so findings based on the whole sample are reported.

### Procedure

The present study was conducted as part of a larger project on attention biases in AN (Radix et al., under review) which comprised three sessions in total. Prior to participation, written informed consent was obtained from all participants (and their parents/legal custodians for participants under 18 years of age) after a comprehensive explanation of the procedures. The task examined in the present study was administered in the first session of the project, whereas additional experimental tasks (which investigated the role of anxiety in triggering attention biases) were delivered in sessions two and three (see Radix et al., under review). The first session of the project began with the diagnostic interview. After that, photographs of the participants’ bodies that were to be used as stimuli in sessions two and three were taken. Then the present task was administered, which assessed attention biases by recording eye movements during passive viewing of ED-related information (pictures of bodies) versus social information (pictures of faces). Participants completed the questionnaire measures between sessions one and two. The study was approved by the ethics committee of the Medical Faculty of the Ruhr-University Bochum (15-5541-BR) as well as the ethics committee of the Medical Faculty of the LMU Munich (Project-No. 814-16). Participants received a reimbursement of €50 for participation in the whole project.

#### Stimuli

Stimuli consisted of photographs of faces and bodies that were presented in grayscale on black background. Face stimuli were taken from the Karolinska Directed Emotional Faces database (KDEF; Lundqvist et al., 1998) and edited so that only the facial area was visible. Pictures of six female models displaying neutral, happy, and angry facial expressions (two models per emotion) were used. Body stimuli were taken from a set of standardized photographs of female bodies of different weight categories in underwear (Horndasch et al., 2015). Pictures of two normalweight (BMI 20.7–21.0), two underweight (BMI 17.0–17.6), and two overweight (BMI 25.2–29.6) bodies in front view were used.

#### Experimental Task

**Fig. 1:**
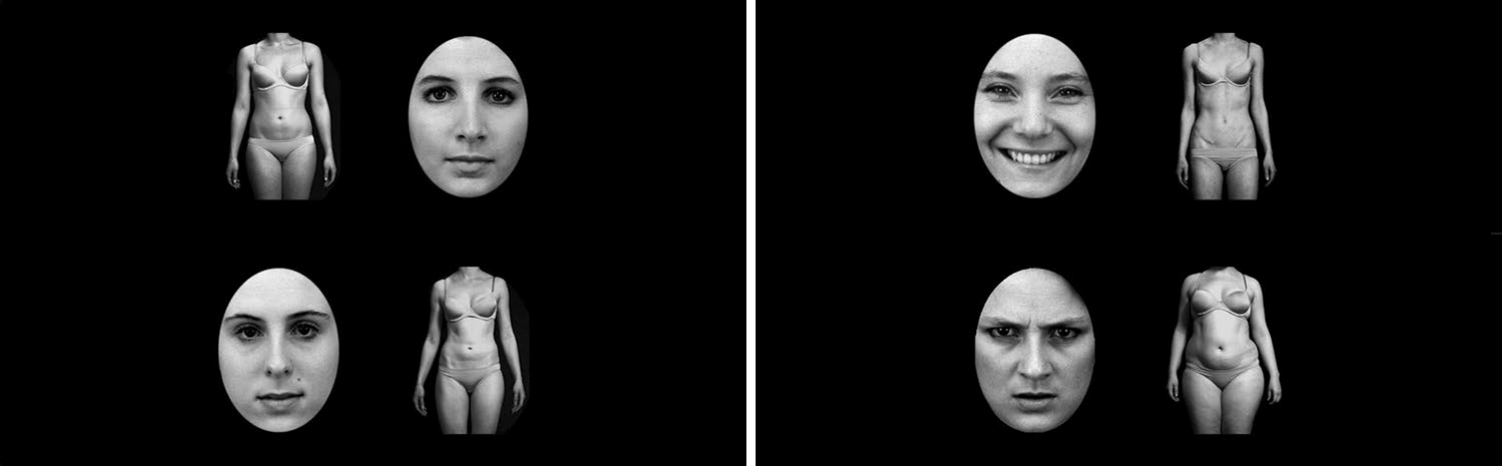
Examples of a neutral trial (left side) and an emotional (right side) trial in the eye-tracking paradigm. Stimuli were taken from Lundqvist et al. (1998) and Horndasch et al. (2015)

Participants were seated in front of a 22-in. monitor (1680 × 1050 pixel resolution; viewing distance approximately 70 cm) on which the experiment was presented using E-Prime Version 2.0 (Psychology Software Tools Inc., 2013). Each trial began with a fixation cross that had to be fixated for 500 ms for the trial to start. Then a 2 × 2 stimulus array was presented for 12 s (cf. Pinhas et al., 2014). The task consisted of 24 neutral trials and 24 emotional trials (conform to the recommended minimum trial number for eye-tracking research; Orquin & Holmqvist, 2018) that were presented in random order. In neutral trials, the stimulus array consisted of two normalweight bodies and two neutral faces. In emotional trials, an underweight body, an overweight body, a happy face, and an angry face were presented (see Fig. 1 for example stimulus displays). In both trial types the position of pictures was randomly assigned to one of the quadrants with each picture category being presented in each quadrant exactly twelve (neutral trials) or six (emotional trials) times and each model being presented equally often. Pictures had a size of 275 × 375 pixels and were presented with a distance of 150 pixels from each other horizontally as well as vertically (equivalent to a size of approximately 7.8 × 10.6 cm / 6.4° × 8.6° visual angle and a distance of 4.2 cm / 3.4° visual angle; i.e., pictures were presented between 1.7° and 8.1° of visual angle horizontally and between 1.7° and 10.3° of visual angle vertically). Participants were instructed to fixate the fixation cross and then freely view the stimuli with the only requirement being that their attention had to remain on the screen.

After the viewing task participants evaluated the face and body stimuli (presented in random order) on the dimensions valence (ranging from 1 = very unpleasant to 9 = very pleasant) and arousal (ranging from 0 = not at all arousing to 9 = very arousing) using the 9-point Self-Assessment Mannequin scale (Lang, 1980). The results of this evaluation are presented in Supplement 2.

#### Eye-Tracking

Eye movements during the experimental task were registered binocularly (but only data of the left eye were analyzed) at a sampling rate of 500 Hz with a monitor-integrated eye-tracking system that used infrared video-based tracking technology (RED500; SensoMotoric Instruments) and iView X software (SensoMotoric Instruments). Before the task started a 9-point calibration and validation procedure was conducted and calibration was accepted if the average error was less than 0.5° of visual angle. Eye movements were detected using a velocity based detection method implemented in SMI BeGaze 3.7 software (SensoMotoric Instruments) with saccades defined as events with a velocity above 75°/s for a minimum duration of 20 ms and fixations defined as events with lower velocities and a minimum duration of 60 ms (Dinkler et al., 2019; Fujiwara et al., 2017). To ensure adequate data quality, line graphs and scan paths of each trial were visually inspected and trials with excessive blinks or a considerable proportion of missing data were excluded. Only participants with at least 12 valid trials for both trial types were included in the final sample. On average, 23.03 neutral trials (*SD* = 2.22) and 23.11 emotional trials (*SD* = 2.14) were available per participant (not different between groups: *F*s ≤ 1.99, *p*s > 0.1).

Two eye-tracking indices were examined: percentage of first fixations on each picture category as an indicator of initial orientation of attention and mean percentage of dwell time (defined as sum of durations of all fixations and saccades that hit the area of interest) on each picture category during the whole duration of the trial as an indicator of maintenance of attention. Reliabilities of these outcome measures were assessed by correlating scores based on odd versus even trials (split-half reliability; see, e.g., Platt et al., 2022, for a similar approach). Split half-reliabilities for percentages of first fixations were acceptable to good (Spearman-Brown-corrected reliability 0.77-0.88) while split half-reliabilities for percentages of dwell time were good to excellent (Spearman-Brown-corrected reliability 0.83-0.95).

### Data Analysis

Statistical data analysis was conducted with SPSS. The eye-tracking indices were analyzed separately for neutral and emotional trials using repeated-measures analyses of variance (ANOVAs) with the within-subjects factor PictureCategory (2 for neutral trials: face, body; 4 for emotional trials: happy face, angry face, underweight body, overweight body) and the between-subjects factor Group (3: AN, MD, HC). Significant effects were followed up by post-hoc ANOVAs and subsequent *t*-tests. Degrees of freedom were adjusted via the Greenhouse–Geisser correction when necessary, i.e., when the assumption of sphericity was violated. For all analyses, the significance level was set to *p* = 0.05 (two-tailed) and effect sizes are reported: *η*_*p*_^*2*^ for ANOVAs (with *η*_*p*_^*2*^ = 0.01 interpreted as a small effect, *η*_*p*_^*2*^ = 0.06 interpreted as a medium effect, and *η*_*p*_^*2*^ = 0.14 interpreted as a large effect; Cohen, 1988) and Cohen’s *d* for *t*-tests (with *d* = 0.20 interpreted as a small effect, *d* = 0.50 interpreted as a medium effect, and *d* = 0.80 interpreted as a large effect; Cohen, 1988).

## Results

### Initial Orientation of Attention

**Fig. 2:**
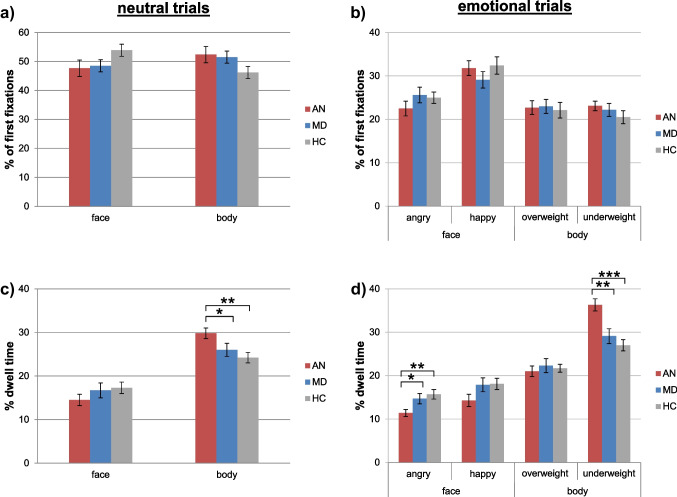
Results of the investigated eye-tracking indices. The top panels show percentage of first fixations on the different picture categories in the neutral trials (**a**) as well the emotional trials (**b**). The bottom panels show percentage of dwell time on the different pictures across the whole trial duration in neural trails (**c**) and emotional trials (**d**). Note that percentage of dwell time in neutral trials is the mean percentage across both pictures of a category, not the sum. Significant group differences are indicated as follows: * *p* < 0.05, ** *p* < 0.01, *** *p* < 0.001. Error bars represent standard errors

For neutral trials, the ANOVA on percentage of first fixations yielded no significant effects (*F*s ≤ 1.94 *p*s > 0.1). For emotional trials, the ANOVA yielded a significant main effect of PictureCategory (*F*_3,207_ = 14.59, *p* < 0.001, *η*_*p*_^*2*^ = 0.18), resulting from participants orienting their attention towards pictures of happy faces significantly more often than towards other pictures (*t*s_71_ ≥ 4.3, *p*s < 0.001), while the PictureCategory × Group interaction was non-significant (*F* < 1). See Fig. 2a, b and Supplementary Table 1 for descriptive results of the eye-tracking data.

### Maintenance of Attention

For neutral trials, the ANOVA on percentage of dwell time yielded a significant main effect of PictureCategory (*F*_1,69_ = 51.01, *p* < 0.001, *η*_*p*_^*2*^ = 0.43) with longer dwell times on pictures of bodies compared to pictures of faces, while the main effect of Group was not significant (*F*_2,69_ = 2.31, *p* > 0.1). Furthermore, a significant PictureCategory × Group interaction (*F*_2,69_ = 3.22, *p* = 0.046, *η*_*p*_^*2*^ = 0.09) emerged, which was followed up by one-way ANOVAs with the factor Group performed separately for faces and bodies and subsequent *t*-tests. For bodies, a significant effect of Group emerged (*F*_2,69_ = 5.33, *p* = 0.007, *η*_*p*_^*2*^ = 0.13), due to adolescents with AN fixating longer on the bodies than adolescents with no mental illness (*t*_50_ = 3.23, *p* = 0.002, *d* = 0.90) and adolescents with MD (*t*_46_ = 2.02, *p* = 0.049, *d* = 0.59; this comparison would not be significant after correction for multiple testing; Holm, 1979). For faces, no effect of Group was found (*F*_2,69_ = 1.17, *p* > 0.1, *η*_*p*_^*2*^ = 0.03).

For emotional trials, a significant main effect of PictureCategory (*F*_1.89,130.20_ = 76.93, *p* < 0.001, *η*_*p*_^*2*^ = 0.53), resulting from significant differences between all categories (*t*s_71_ ≥ 3.67, *p*s < 0.001), and a significant PictureCategory × Group interaction (*F*_3.77,130.20_ = 5.75, *p* < 0.001, *η*_*p*_^*2*^ = 0.14) emerged, while the main effect of Group was non-significant (*F* < 1). The interaction was followed up by one-way ANOVAs with the factor Group performed separately for each picture category and subsequent *t*-tests. A significant effect of Group emerged for angry faces (*F*_2,69_ = 5.31, *p* = 0.007, *η*_*p*_^*2*^ = 0.13) as well as underweight bodies (*F*_2,69_ = 12.40, *p* < 0.001, *η*_*p*_^*2*^ = 0.26), due to the adolescents with AN fixating longer on underweight bodies and shorter on angry faces than adolescents with no mental illness (underweight bodies: *t*_50_ = 4.86, *p* < 0.001, *d* = 1.35; angry faces: *t*_50_ = 3.17, *p* = 0.003, *d* = 0.88) and adolescents with MD (underweight bodies: *t*_46_ = 3.35, *p* = 0.002, *d* = 0.98; angry faces: *t*_46_ = 2.35, *p* = 0.023, *d* = 0.69). No effects emerged for happy faces (*F*_2,69_ = 2.41, *p* = 0.097, *η*_*p*_^*2*^ = 0.07) as well as overweight bodies (*F* < 1). Results are presented in Fig. 2c, d as well as Supplementary Table 1.

In addition to the planned analyses that examined our a priori hypotheses, we conducted post-hoc exploratory analyses to determine whether groups differed in the course of attention deployment over time: We split the trials into four 3-s time intervals and conducted TimeWindow × PictureCategory × Group ANOVAs on percentage of dwell time. The results indicated that in the neutral trials, differences in dwell time resulted from the group with AN dwelling more on bodies particularly during the middle part of the trial (see Supplement 3 for detailed results).

### Additional Analyses

As participants in the three groups differed in age and IQ, we computed Pearson's correlations between these variables and percentage of dwell time on normalweight bodies, underweight bodies, and angry faces, to investigate if these participant characteristics may have accounted for the group differences we found. Correlations were computed separately within each group to rule out that emerging correlations would be artefacts of the group differences in both variables. Significant correlations between age and dwell time on underweight bodies (*r* = -0.47, *p* = 0.020) and angry faces (*r* = 0.60, *p* = 0.002) emerged only within the group of adolescents with no mental illness, indicating no systematic relationships.

## Discussion

The aim of the present study was to investigate attention biases for disorder-related stimuli (i.e., pictures of bodies) versus social stimuli (i.e., pictures of faces) in adolescents with AN compared to adolescents with MD or no mental illness using a passive-viewing eye-tracking paradigm where both types of stimuli were presented simultaneously and directly competed for attention. We found more pronounced biases in the maintenance of attention on body pictures in adolescents with AN than in the comparison groups, particularly for pictures of underweight bodies, at the expense of looking less at social stimuli.

Adolescents in all groups looked longer at bodies than at faces, i.e., showed attention biases for bodies in maintenance of attention in both, neutral and emotional trials. This is in line with results from another task in an overlapping sample (Radix et al., under review) and other studies finding attention biases for stimuli related to body shape in female adolescents as young as 14 years (Green & McKenna, 1993). It might reflect the high importance of body and shape for female adolescents in general (Wadden et al., 1991). In the emotional trials, all participants preferentially looked at underweight bodies. This is in line with Watson et al. (2010), who found that when pictures of underweight females were presented, both, women with and without AN, spent less time looking at the females’ faces. Our result also corresponds with findings from Radix et al. (under review) and is in line with other studies that have found attention biases towards thin versus overweight bodies in young women (Glauert et al., 2010; Joseph et al., 2016). However, other studies have not reported such main effects of picture category but found only interactions of stimulus category and body dissatisfaction, i.e., different attention biases in women with high and low body dissatisfaction (Moussally et al., 2016; Tobin et al., 2018).

It has to be noted that there might also be another plausible explanation for the preferential looking at bodies across all groups and both trial types. Body and face stimuli differed in physical properties that might influence viewing times: Face stimuli had a higher luminance while body stimuli were more complex and included more details to explore and were presumably more interesting than the face stimuli. Therefore, the face stimuli might have become boring earlier as only two pictures per category were presented and repeated several times. The preference to look at underweight bodies in the emotional trials, however, cannot be explained by these physical properties of the stimuli as pictures of underweight and overweight bodies had similar complexity and luminance.

In line with our hypotheses, adolescents with AN looked longer at bodies than adolescents in the comparison groups, i.e., showed stronger attention biases for body pictures. This was the case in both, neutral as well as emotional trials, where the AN group’s preference for bodies was particularly pronounced for underweight body pictures. Finding more pronounced attention biases for bodies in adolescents with AN across both trial types together with the medium to large between-group effect sizes (*d* = 0.59–1.35) underline the robustness of this result. Importantly, adolescents with AN differed from both, adolescents with no mental illness as well as adolescents with MD, indicating that the more pronounced attention biases for body-related information are specific to their ED and cannot be attributed to comorbid depression, anxiety, or more general psychopathology.^3^ Our results are consistent with those of previous studies investigating attention biases for body-related in comparison to socially relevant stimuli that also found adolescents (Pinhas et al., 2014) and adults (Watson et al., 2010) with AN to show attention biases towards bodies and less attendance of socially relevant information. However, one study (Cornelissen et al., 2016) found women with AN to look more at the face region than women with no mental illness in a body-size estimation task where full body images including the head/face were presented. Our results are also in line with cognitive-behavioral theories predicting biases for body weight and shape related information in individuals with AN (Williamson et al., 1999, 2004). According to these theories, these biases could *directly* contribute to the maintenance of the disorder by reinforcing maladaptive schemata related to weight and shape which fuel ED psychopathology.

The particularly pronounced attention bias for underweight body pictures is also in line with our expectations and replicated the result of Pinhas et al. (2014). It was also observed using a different paradigm in an overlapping sample (Radix et al., under review). Preferential looking at thin bodies has also been reported for other EDs: Blechert and colleagues (2009) found adult women with bulimia nervosa to fixate longer on low BMI bodies and shorter on high BMI bodies than women with no mental illness. By contrast, Rieger et al. (1998) found an attention bias away from words describing a thin physique in women with EDs. The preference for thin bodies and the dislike of overweight bodies in AN is also reflected in explicit evaluations of body pictures: most studies (including the present one, see Supplement 2) found adolescents and adults with AN to evaluate bodies with higher BMIs more negatively and bodies with lower BMIs more positively than females with no mental illness (Horndasch et al., 2015, 2018; von Wietersheim et al., 2012; but see also Watson et al., 2010). Underweight bodies are consistent with the goals of (most) individuals with AN and looking at them could motivate the maintenance of weight loss behaviors (Mento et al., 2021; Norris et al., 2006; Pinhas et al., 2014), so that biases for underweight bodies could have a particularly detrimental direct effect on ED psychopathology.

The aforementioned higher complexity of the body stimuli compared to the face stimuli could provide an alternative explanation as to why adolescents with AN showed a stronger preference for bodies vs. faces than the comparison groups: Adults (Lang et al., 2014) as well as adolescents (Lang & Tchanturia, 2014) with AN have been found to show weaker central coherence, i.e., superior attention to detail alongside poorer holistic processing, compared to individuals with no mental illness. Hence, the preferential attention for bodies (the stimuli characterized by more details), could reflect a bias for preferential processing of detail rather than preferential processing of stimuli related to weight and shape. Importantly, the physical properties of the stimuli cannot explain the particularly pronounced attention bias for underweight bodies in the emotional trials, in which underweight body pictures were presented alongside overweight body pictures. These two stimulus categories did not vary in complexity but only in the weight of the depicted bodies, hence, one would expect adolescents with AN to show increased dwell time also for overweight body pictures if effects were explained only by increased attention to detail in this group. Since this was not the case, the results seem to be at least to some extent modulated by picture content.

Importantly, no differences in initial orientation of attention were found between the three groups. Instead, participants in all groups showed preferential orientation towards happy faces in the emotional trials, which is in line with a meta-analysis showing that biases for positive stimuli occur in early attentional processing (Pool et al., 2016). Also, our exploratory analysis that examined the course of visual attention over time did not indicate more pronounced group differences in dwell time at the beginning of the trials. Thus, we found no evidence that adolescents with AN first show hypervigilance and then avoidance of body pictures. While initial orientation of attention is a more automatic and bottom-up controlled component of attention, maintenance of attention is more top-down controlled (Theeuwes et al., 2000), especially in such a task like the passive-viewing task employed in the present study, in which participants can deliberately choose where to look. Hence, our results suggest that adolescents with AN show more pronounced attention biases for disorder-relevant information (versus social information) in an internally-controlled and at least partially conscious aspect of attention, while not differing from the comparison groups in more automatic aspects of attention. This is similar to results from Kerr-Gaffney et al. (2021) who found individuals with AN to show initial orientation towards social stimuli but disengage from such stimuli more quickly than individuals with no mental illness.

Our results indicate that adolescents with AN do not only show attention biases for body stimuli when these are presented together with neutral stimuli but also in the presence of salient social information as pictures of emotional faces. In return for looking more at bodies, adolescents with AN attended less to faces, particularly to faces showing angry expressions. This is partly comparable with results in individuals with social anxiety disorder who have been found to show attention biases *towards* angry faces in early attentional processes (Armstrong & Olatunji, 2012; Duval et al., 2020; Liang et al., 2017) and avoidance of (angry) faces in later, more conscious aspects of attention (Garner et al., 2006), as well as gaze (Weeks et al., 2013) and behavior (Heuer et al., 2007). Importantly, this behavioral avoidance might further exacerbate symptoms of social anxiety (Kashdan et al., 2014), thereby acting as a maintenance factor. Less looking at faces and more looking at non-social cues (Frazier et al., 2017) as well as attention biases away from angry faces (García-Blanco et al., 2017; Ghosn et al., 2019) have also been found in individuals with autism spectrum disorders where they seem to be related to poorer social adjustment (Klin et al., 2002) and communication difficulties (García-Blanco et al., 2017). By attending less to social information, important social cues that are a key to successful communication and interaction are likely to be missed, which might contribute to the development and amplification of interpersonal difficulties (Kerr-Gaffney et al., 2019, 2021). The avoidance of angry faces, in particular, might involve behavioral avoidance of potentially aversive social interactions, thereby hampering the ability to adaptively solve interpersonal conflicts (García-Blanco et al., 2017). Through these mechanisms, the biases found in the present study could have an additional *indirect* negative effect on ED psychopathology. Of note, it has been suggested that different mechanisms may explain reduced attention to social cues in AN and autism spectrum disorders (Kerr-Gaffney et al., 2022). The present study might help to shed light on these different mechanisms as it indicates that increased attention to disorder-related characteristics of people (i.e., their bodies) might underlie the reduced attention to their faces in individuals with AN while probably other mechanisms (as for example attempts to reduce overstimulation or stress; Kerr-Gaffney et al., 2022) are responsible for the reduced attention to faces in individuals with autism spectrum disorders. However, in the present study pictures of faces were always presented alongside pictures of bodies (i.e., highly relevant disorder-related stimuli) so it remains unknown whether adolescents with AN would also look less at faces in the absence of disorder-related information (as found in adults with AN: Fujiwara et al., 2017; Kerr-Gaffney et al., 2^021^).

We designed the present study not only to gain insight in the preferential processing of disorder-related information in individuals with AN, but also hoping that the investigation of disorder-related information in relation to socially relevant information would help to understand the altered attentional processing of social cues in AN. The finding that adolescents with AN direct more attention to body related stimuli at the expense of directing less attention to emotional faces is indeed in line with our assumptions. However, previous studies investigating processing of social cues using event-related potentials (Hatch et al., 2010; Sfärlea et al., 2016) or functional magnetic resonance imaging (Fonville et al., 2014) suggested alterations in early, automatic stages of processing of emotional faces, whereas the present study did not find alterations in early, automatic attention allocation but in later, more conscious attentional processing. Hence, further investigation of the automatic and conscious processes involved in the processing of ED-related and socially relevant cues in AN are necessary to understand their interplay.

### Strengths and Limitations

The present study has several strengths. Some of them concern the sample: In contrast to previous studies, we compared adolescents with AN not only to adolescents with no mental illness but also to a clinical comparison group of adolescents with MD, allowing us to draw conclusions about biases being specific for adolescents with AN. To ensure diagnostic accuracy, all participants underwent an extensive diagnostic assessment using a standardized interview instead of relying on self-reported diagnoses. Although the size of our sample is modest, it is still considerably larger than in the previous studies investigating attention biases for ED-related versus social stimuli (Pinhas et al., 2014; Watson et al., 2010). Another strength is that we determined the reliability of our outcome measures which was acceptable to excellent for both eye-tracking indices, further underlining the robustness of our results.

Some limitations have to be noted as well. As mentioned earlier, the present study was conducted as part of a larger project on attention biases in adolescents with AN (Radix et al., under review) and photographs of the participants’ bodies that were to be used as stimuli in another task were taken in the same session as the task described in the present study was administered. This might have activated body related schemata (Labarge et al., 1998) which could have contributed to participants attending more to bodies. It remains unclear if the same effects would have been found if the task was administered completely independently from other procedures. However, as the procedure was the same for all participants, it is unlikely that it provides an explanation for the group differences we found.

Another limitation relates to our stimuli: we used standardized stimuli with body and face stimuli taken from validated databases (Horndasch et al., 2015; Lundqvist et al., 1998), presented in grayscale, and showing no distracting features like different haircuts or clothes, thereby eliminating as many confounding characteristics as possible. Still, pictures of faces and bodies varied in luminance and complexity and it cannot be ruled out that physical properties of the pictures in addition to picture content influenced our results. However, as pictures of faces and bodies are innately different in complexity this limitation does not only apply to our study but is a general limitation when stimuli of different categories compete for attention.

Further limitations concern our study sample: i) The three groups differed significantly in age and IQ, with participants in the AN group being significantly younger and having a higher IQ than those in the comparison groups. However, as neither age nor IQ were systematically related to our outcome measures, it is unlikely that group differences in dwell times can be attributed to variations in these participant characteristics. ii) We have only limited demographic data on the participants and cannot provide information on race/ethnicity and socio-economic status. It is therefore possible that our results apply only to a certain subgroup of AN patients, calling the generalizability of our study results into question. iii) The considerable overlap in psychopathology symptom scores between the two clinical groups, with not only the group with AN reporting high depression symptoms (which is expected) but also the group with MD reporting high ED psychopathology (see Table 1), calls the precision of the diagnostic assessment into question, even though we administered a well-established standardized interview. Unfortunately, the interrater-reliability for the diagnostic interview could not be determined in the present study as the diagnostic interviews were not audiotaped.

It also has to be mentioned that our study design which aimed to investigate attention biases for disorder-relevant information *versus* social information by presenting pictures of bodies alongside pictures of faces, is not only a strength of the present study as it taps into a gap in the literature, but also entails limitations: It does not allow to draw conclusions about attention biases for faces or bodies in the absence of the other category or to differentiate between biases towards underweight bodies and away from angry faces in adolescents with AN.

### Clinical Implications

We found adolescents with AN (in comparison to adolescents with MD or adolescents with no mental illness) to show more pronounced attention biases towards ED-related information, i.e., pictures or bodies, at the expense of looking less at socially relevant information, i.e., pictures of faces. These biases for disorder-related versus socially relevant information might be a starting-point for a “dual” cognitive bias modification approach: on the one hand, training individuals with AN to direct their attention away from ED-related information might have a direct positive influence on their ED symptoms, by reducing the impact of maladaptive weight and shape related schemata. On the other hand, training individuals with AN to direct their attention towards social stimuli might improve their socio-emotional functioning and reduce interpersonal difficulties, thereby indirectly positively influencing ED psychopathology.

Our findings could inform and be integrated into existing evidence-based treatments for adolescents with AN, such as family-based treatment or enhanced cognitive behavioral therapy (Dalle Grave et al., 2019; Le Grange et al., 2022), in multiple ways: For example, knowledge about attention biases might help adolescents with AN to identify and understand the mechanisms that maintain their ED psychopathology, which is a major goal of enhanced cognitive behavioral therapy (Dalle Grave et al., 2019). Therapists may measure their patients’ attention biases towards ED-related information and show them their individual eye-tracking trajectories, thereby illustrating an otherwise abstract process that reinforces dysfunctional cognitive schemata. This might even work without an eye-tracker: The therapist could present the patient multiple (sufficiently large) pictures on paper and videotape them while viewing these, so that the direction of gaze is visible and can be fed back to the patient. Attention bias modification trainings aiming to reduce this bias could, in a next step, help to disrupt the maintaining mechanism. Attention bias modification trainings to increase attention towards social stimuli, on the other hand, could be integrated in the additional module of enhanced cognitive behavioral therapy (that addresses interpersonal difficulties in patients for whom such difficulties were identified as maintaining factors; Dalle Grave & Calugi, 2020) or in Phase III of family-based treatment (which focuses on general issues of adolescent development; Lock & Le Grange, 2012). Training adolescents to direct their attention towards social stimuli including angry faces might promote adaptive solving of interpersonal conflicts, alleviate interpersonal difficulties and help adolescents to establish developmentally appropriate relationships not only within but also outside their families. Learning to tolerate others’ angry facial expression instead of avoiding them could also help to reduce experiential avoidance and to build distress tolerance, which are goals of emotion-focused treatments for individuals with AN (Sala et al., 2016).

## Conclusions

The present study contributes to the scarce literature investigating attention biases for disorder-related versus socially relevant information in AN. While all groups of adolescents showed attention biases for body stimuli, these biases were particularly pronounced in adolescents with AN. In turn, adolescents with AN looked less at pictures of faces. This might have a twofold negative impact on AN psychopathology: the increased attention to ED-related information might have a direct negative influence on ED symptoms while the less attending to social information might have an indirect negative influence through the exacerbation of interpersonal difficulties. Even though this might be a promising avenue for cognitive bias modification approaches, we clearly need further research to understand the interplay between biases in the processing of ED-related information and social information and how this altered processing contributes to ED psychopathology.

## Supplementary Information

Below is the link to the electronic supplementary material.

Supplementary file1 (DOCX 36 KB)

## Footnotes

1 Anxiety is also a frequent comorbid condition in AN (Swanson et al., 2011) and is also related to attention biases (Lau & Waters, 2017). However, adolescents with MD are also expected to show comorbid anxiety disorders frequently (Essau, 2008), so including a group with MD as clinical comparison group allowed us to include a group with anxiety levels presumably comparable to those of the group with AN while at the same time taking into account depression symptoms.

2 A total of 75 participants were tested but three had to be excluded due to low quality of the eye-tracking data (see below for criteria).

3 Of note, investigating relationships between dimensional measures of psychopathology (i.e., questionnaire scores) and attention biases was beyond the scope of the present study and remains the subject of future research.
