# Supplementary material for: Attention Biases for Eating Disorder-Related Stimuli Versus Social Stimuli in Adolescents with Anorexia Nervosa – An Eye-Tracking Study
Source: Res Child Adolesc Psychopathol. 2022 Nov 23;51(4):541–55. doi: 10.1007/s10802-022-00993-3 (PMC10017650; doi:10.1007/s10802-022-00993-3)
Supplement: Supplementary file 1 — Supplementary file1 (DOCX 36 KB) [file 10802_2022_993_MOESM1_ESM.docx]

**SUPPLEMENTARY MATERIAL**

**Supplement 1: Descriptive results of the eye-tracking data**

**Supplementary Table 1:** Descriptive results of the eye-tracking data

|  | **AN** | | **MD** | | **HC** | |
| --- | --- | --- | --- | --- | --- | --- |
|  | % of first fixations | % dwell time | % of first fixations | % dwell time | % of first fixations | % dwell time |
|  | *M (SD)* | *M (SD)* | *M (SD)* | *M (SD)* | *M (SD)* | *M (SD)* |
| *neutral trials* | | | | | | |
| neutral face | 47.7 (14.6) | 14.5 (6.8) | 48.8 (9.2) | 16.7 (7.5) | 53.9 (10.4) | 17.3 (6.5) |
| normalweight body | 52.4 (14.6) | 29.8 (6.5) | 51.5 (9.2) | 26.0 (6.7) | 46.2 (10.4) | 24.2 (6.1) |
| *emotional trials* | | | | | | |
| angry face | 22.5 (8.9) | 11.4 (4.4) | 25.6 (7.9) | 14.7 (5.2) | 25.0 (6.6) | 15.7 (5.3) |
| happy face | 31.8 (8.9) | 14.3 (7.5) | 29.1 (8.7) | 17.9 (7.0) | 32.4 (9.7) | 18.1 (6.3) |
| overweight body | 22.7 (8.4) | 21.0 (6.5) | 23.0 (7.2) | 22.3 (7.0) | 22.1 (8.7) | 21.7 (4.6) |
| underweight body | 23.1 (5.8) | 36.3 (7.2) | 22.2 (6.7) | 29.1 (7.5) | 20.5 (8.7) | 27.0 (6.4) |
| AN = anorexia nervosa; MD = major depression; HC = heathy control; *M* = mean; *SD* = standard deviation | | | | | | |

**Supplement 2: Explicit evaluation of the stimuli**

**Methods**

After the viewing task participants were supposed to evaluate the face and body stimuli (presented in random order) on the dimensions valence (ranging from 1 = very unpleasant to 9 = very pleasant) and arousal (ranging from 0 = not at all arousing to 9 = very arousing) using the 9-point Self-Assessment Mannequin scale (Lang, 1980). To analyze these valence and arousal ratings, PictureCategory (6: happy face, neutral face, angry face, underweight body, normalweight body, overweight body) × Group (3) ANOVAs were performed.

**Results**

Descriptive results are presented in Supplementary Table 2. From the ANOVA on valence ratings, a significant main effect of PictureCategory (*F*_3.4,300.3_ = 90.9, *p* < .001, *η_p_²* = .57) as we as significant main effect of Group (*F*_2,69_ = 16.1, *p* < .001, *η_p_²* = .32) emerged and were further qualified by a significant PictureCategory × Group interaction (*F*_8.7,300.3_ = 3.7, *p* < .001, *η_p_²* = .10). The main effect of PictureCategory resulted from significant differences between all categories (*t*s_71_ ≥ 2.7, *p*s ≤ .009) except between neutral faces and normalweight (*t*_71_ = 1.3, *p* > .1) as well as underweight bodies (*t*_71_ = 1.6, *p* > .1). To follow-up the interaction, post-hoc ANOVAs with the factor Group were calculated for each picture category. For happy faces, the ANOVA yielded a significant effect of Group (*F*_2,69_ = 6.1, *p* = .004, *η_p_²* = .15), indicating that the HC group rated the happy faces more positive than the AN (*t*_37.1_ = 3.6, *p* = .001, *d* = 1.0) and MD groups (*t*_27.1_ = 2.7, *p* = .011, *d* = 0.8). For normalweight and overweight bodies, the significant effects of Group (normalweight bodies: *F*_2,69_ = 16.5, *p* < .001, *η_p_²* = .32; overweight bodies: *F*_2,69_ = 13.5, *p* < .001, *η_p_²* = .281) indicated that the AN group rated these bodies more negative than HC (normalweight bodies: *t*_50_ = 4.7, *p* < .001, *d* = 1.3; overweight bodies: *t*_50_ = 4.1, *p* < .001, *d* = 1.2) and MD groups (normalweight bodies: *t*_46_ = 4.7, *p* < .001, *d* = 1.4; overweight bodies: *t*_46_ = 4.4, *p* < .001, *d* = 1.3). For neutral and angry faces as well as underweight bodies, the post-hoc ANOVAs yielded no effects (*F*s ≤ 2.5 *p*s ≥ .088).

The ANOVA on arousal ratings yielded a significant main effect of of PictureCategory (*F*_3.6,250.2_ = 10.3, *p* < .001, *η_p_²* = .13) resulting from neutral faces being rated lass arousing than all other categories (*t*s_71_ ≥ 4.3, *p*s < .001) and underweight bodies being rated more arousing than normalweight bodies (*t*_71_ = 4.8, *p* < .001). The main effect of Group (*F* < 1) as well as the PictureCategory × Group interaction (*F*_7.3,250.2_ = 1.8, *p* = .081, *η_p_²* = .05) were non-significant.

**Supplementary Table 2.** Descriptive results of the evaluation of the stimuli.

|  | **AN** | **MD** | **HC** |
| --- | --- | --- | --- |
|  | *n* = 28 | *n* = 20 | *n* = 24 |
|  | *M* (*SD*) | *M* (*SD*) | *M* (*SD*) |
| *Valence* |  |  |  |
| angry face | 2.3 (1.1) | 2.8 (1.7) | 2.9 (1.3) |
| neutral face | 4.9 (1.2) | 5.4 (1.5) | 5.8 (1.5) |
| happy face | 6.8 (1.9)^a^ | 7.2 (1.5)^a^ | 8.2 (0.8)^b^ |
| overweight body | 2.9 (1.8)^a^ | 5.1 (1.6)^b^ | 4.9 (1.5)^b^ |
| normalweight body | 4.5 (1.6)^a^ | 6.5 (1.2)^b^ | 6.4 (1.4)^b^ |
| underweight body | 5.0 (1.8) | 4.8 (1.2) | 5.1 (1.3) |
| *Arousal* |  |  |  |
| angry face | 3.2 (2.1) | 4.1 (1.9) | 4.8 (2.4) |
| neutral face | 2.5 (1.8) | 3.1 (1.7) | 2.7 (1.4) |
| happy face | 3.5 (2.2) | 4.8 (2.2) | 4.1 (2.5) |
| overweight body | 4.2 (2.7) | 3.7 (2.0) | 4.0 (1.7) |
| normalweight body | 3.5 (1.9) | 3.9 (2.3) | 3.3 (1.5) |
| underweight body | 4.8 (2.5) | 4.6 (1.9) | 4.4 (1.7) |
| AN = anorexia nervosa; MD = major depression; HC = heathy control; *M* = mean; *SD* = standard deviation; ^a,b^ groups with different superscript letters differed signiﬁcantly. | | | |

**Discussion**

The preference for thin bodies across all groups was not reflected in the explicit evaluation of the stimuli where the following pattern was found: happy faces > neutral faces = normalweight bodies = underweight bodies > overweight bodies > angry faces. So participants looked longer at bodies despite rating faces as equally (neutral faces compared to normalweight bodies) or even more pleasant (happy faces compared to under- and overweight bodies) than bodies. The more negative evaluation of overweight compared to normalweight bodies across all groups is in line with studies finding more negative evaluation of overweight compared to normalweight/slim bodies explicitly as well as implicitly in unselected adults as well as adolescents (e.g., Baluch et al., 1997; Schupp & Renner, 2011).

The particularly pronounced preference for underweight bodies in AN, however, was reflected in the explicit evaluation of the stimuli: Girls with AN evaluated overweight and normalweight bodies more negatively than the control groups, which is in line with previous research in adolescents as well as adults that found AN patients to evaluate bodies with higher BMIs more negatively (e.g., more unpleasant, more aversive, less attractive) and bodies with lower BMIs more positively (e.g., more pleasant, more attractive) than healthy girls or women (e.g., Baluch et al., 1997; Cserjési et al., 2010; George et al., 2011; Horndasch et al., 2015; 2018; Spring & Bulik, 2014; von Wietersheim et al., 2012; but see also Watson et al., 2010).

**Supplement 3: Exploratory investigation differences in dwell time over time**

Percentage of dwell time was reanalyzed by splitting the trial into four 3-second time intervals and conducting TimeWindow (4: time window 1-4) × PictureCategory (2 for neutral trials and 4 for emotional trials) × Group (3) ANOVAs.

For neutral trials, this analysis revealed significant main effects of TimeWindow (*F*_2.3,161.0_ = 32.0, *p* < .001, *η_p_²* = 317) and PictureCategory (*F*_1,69_ = 54.6, *p* < .001, *η_p_²* = 442), while the main effect of Group was not significant (*F*_2,69_ = 2.6, *p* = .082). Furthermore, significant interactions of PictureCategory × Group (*F*_2,69_ = 53.3, *p* = .042, *η_p_²* = .088), TimeWindow × PictureCategory (*F*_3,207_ = 5.4, *p* = .001, *η_p_²* = .072), and TimeWindow × PictureCategory × Group (*F*_6,207_ = 2.9, *p* = .010, *η_p_²* = .078) emerged. To follow up the 3-way interaction, PictureCategory × Group ANOVAs were calculated separately for each time window. Main effects of PictureCategory emerged in each time window (*F*s ≥ 23.6, *p*s < .001, *η_p_²*s ≥ .255), main effects of Group emerged in time windows 2 (*F*_2,69_ = 4.9, *p* = .010, *η_p_²* = .124; not interpretable due to an interaction) and 4 (*F*_2,69_ = 3.6, *p* = .033, *η_p_²* = .094; descriptively both clinical groups dwelled longer at pictures than the HC group but these group differences not significant after Bonferroni-Holm correction), and interactions emerged in time windows 1 (*F*_2,69_ = 3.4, *p* = .038, *η_p_²* = .091), 2 (*F*_2,69_ = 3.9, *p* = .026, *η_p_²* = .101), and 3 (*F*_2,69_ = 5.0, *p* = .010, *η_p_²* = .126). The interactions were followed up by one-way ANOVAs with the factor Group performed separately for faces and bodies and subsequent *t*-tests. In time window 1, none of the one-way ANOVAs tuned our significant (bodies: *F*_2,69_ = 1.9, *p* > .1; faces: *F*_2,69_ = 2.6, *p* = .082). In time window 2, a significant main effect of Group emerged for bodies (*F*_2,69_ = 7.3, *p* = .001, *η_p_²* = .174), due to the AN group looking longer at bodies compared to the HC group (*t*_50_ = 3.8, *p* < .001, *d* = 1.1; difference between AN and MD groups not significant after Bonferroni-Holm correction). For faces, no effect of Group was found (*F* < 1). In time window 3, a significant main effect of Group emerged for bodies (*F*_2,69_ = 5.0, *p* = .009, *η_p_²* = .127, again due to the AN group looking longer at bodies compared to the HC (*t*_50_ = 2.4, *p* = .020, *d* = 0.7) and MD groups (*t*_46_ = 2.8, *p* = .007, *d* = 0.8). For faces, no effect of Group was found (*F*_2,69_ = 3.1, *p* = .052). Supplementary Figure 1 illustrates the time-dependency of group differences in dwell time on normalweight body pictures:

***Supplementary Figure 1:*** Percentage of dwell time on normalweight bodies in neutral over the course of time, i.e., splitted into four 3-second time windows.

For emotional trials, the TimeWindow (4) × PictureCategory (4) × Group (3) ANOVA revealed significant main effects of TimeWindow (*F*_3,207_ = 109.2, *p* < .001, *η_p_²* = .613) and PictureCategory (*F*_1.9,131.5_ = 79.6, *p* < .001, *η_p_²* = .536), while the main effect of Group was not significant (*F* < 1). In addition, significant PictureCategory × Group (*F*_3.8,131.5_ = 5.8, *p* < .001, *η_p_²* = .144) and TimeWindow × PictureCategory (*F*_6.0,410.6_ = 4.5, *p* < .001, *η_p_²* = .061) interactions emerged. However, as the 3-way interaction of TimeWindow, PictureCategory, and Group was not significant (*F* < 1), the effects were not further followed up.

**Supplement References**

Baluch, B., Furnham, A., & Huszcza, A. (1997). Perception of body shapes by anorexics and mature and teenage females. *Journal of Clinical Psychology, 53*(2), 167-175.

Cserjési, R., Vermeulen, N., Luminet, O., Marechal, C., Nef, F., Simon, Y., & Lénárd, L. (2010). Explicit vs. implicit body image evaluation in restrictive anorexia nervosa. *Psychiatry Research*, 175(1-2), 148-153.

George, H.R., Cornelissen, P.L., Hancock, P.J., Kiviniemi, V.V., & Tovee, M.J. (2011). Differences in eye‐movement patterns between anorexic and control observers when judging body size and attractiveness. *British Journal of Psychology, 102*(3), 340-354.

Horndasch, S., Heinrich, H., Kratz, O., Mai, S., Graap, H., & Moll, G.H. (2015). Perception and evaluation of women’s bodies in adolescents and adults with anorexia nervosa. *European Archives of Psychiatry and Clinical Neuroscience, 265*(8), 677-687.

Horndasch, S., Kratz, O., Van Doren, J., Graap, H., Kramer, R., Moll, G.H., & Heinrich, H. (2018). Cue reactivity towards bodies in anorexia nervosa–common and differential effects in adolescents and adults. *Psychological Medicine, 48*(3), 508-518.

Lang, P.J. (1980). Behavioral treatment and bio-behavioral assessment: Computer applications. In J. B. Sidowski, J. H. Johnson & T. A. Williams (Eds.), *Technology in Mental Health Care Delivery Systems* (pp. 119-137). Norwood: Ablex.

Schupp, H.T., & Renner, B. (2011). The implicit nature of the anti-fat bias. *Frontiers in Human Neuroscience*, 5: 23.

Spring, V.L., & Bulik, C.M. (2014). Implicit and explicit affect toward food and weight stimuli in anorexia nervosa. *Eating Behaviors, 15*(1), 91-94.

von Wietersheim, J., Kunzl, F., Hoffmann, H., Glaub, J., Rottler, E., & Traue, H.C. (2012). Selective attention of patients with anorexia nervosa while looking at pictures of their own body and the bodies of others: an exploratory study. *Psychosomatic Medicine, 7*4(1), 107-113.

Watson, K.K., Werling, D.M., Zucker, N.L., & Platt, M.L. (2010). Altered social reward and attention in anorexia nervosa. *Frontiers in Psychology*, 1, 36.
